# Performance Sensing Data Prediction for an Aircraft Auxiliary Power Unit Using the Optimized Extreme Learning Machine †

**DOI:** 10.3390/s19183935

**Published:** 2019-09-12

**Authors:** Xiaolei Liu, Liansheng Liu, Lulu Wang, Qing Guo, Xiyuan Peng

**Affiliations:** 1 School of Electronics and Information Engineering, Harbin Institute of Technology, Harbin 150080, China; lluxiaolei@hit.edu.cn (X.L.); qguo@hit.edu.cn (Q.G.); 2 China Southern Airlines Company Limited Shenyang Maintenance Base, Shenyang 110169, China; wanglulu@csair.com; 3 China Southern Airlines Engineering Technology Research Center, Shenyang 110169, China

**Keywords:** auxiliary power unit, improved neural network, stable prediction, performance sensing data prediction

## Abstract

The aircraft auxiliary power unit (APU) is responsible for environmental control in the cabin and the main engines starting the aircraft. The prediction of its performance sensing data is significant for condition-based maintenance. As a complex system, its performance sensing data have a typically nonlinear feature. In order to monitor this process, a model with strong nonlinear fitting ability needs to be formulated. A neural network has advantages of solving a nonlinear problem. Compared with the traditional back propagation neural network algorithm, an extreme learning machine (ELM) has features of a faster learning speed and better generalization performance. To enhance the training of the neural network with a back propagation algorithm, an ELM is employed to predict the performance sensing data of the APU in this study. However, the randomly generated weights and thresholds of the ELM often may result in unstable prediction results. To address this problem, a restricted Boltzmann machine (RBM) is utilized to optimize the ELM. In this way, a stable performance parameter prediction model of the APU can be obtained and better performance parameter prediction results can be achieved. The proposed method is evaluated by the real APU sensing data of China Southern Airlines Company Limited Shenyang Maintenance Base. Experimental results show that the optimized ELM with an RBM is more stable and can obtain more accurate prediction results.

## 1. Introduction

The aircraft auxiliary power unit (APU) is designed to provide power for the aircraft independently [1]. In fact, the core of the APU is a small gas turbine engine providing power and compressed air [2,3]. Before the aircraft takes off, the APU provides power for lighting and air conditioning in the cabin, and also provides the compressed air for starting the main engines of the aircraft. After climbing to a certain height, the APU is shut down. When an emergency situation occurs (e.g., fault of the main engine), the APU can be started again to help restart the main engines. After landing, the APU supplies power for lighting and air conditioning again. In this way, the main engines can be turned off earlier to save fuel and reduce noise and emissions.

The cost of APU maintenance and overhaul is relatively high. If the cost can be reduced, the economic benefits of airlines can be greatly improved [4,5]. The maintenance of APUs by airlines is mainly under the form of regular maintenance and maintenance after failure. In this way, the APU often works in the sub-health state and is not repaired until the APU fails to work, which not only increases the economic burden of airlines, but also threatens flight safety. To enhance the reliability of the APU and reduce the economic cost for airlines, prognostics and health management (PHM) is an effective strategy [6,7]. PHM has strong theoretical and engineering research demand for condition-based maintenance. PHM can provide effective information support for failure warning [8], anomaly detection [9,10], remaining useful life (RUL) prediction [11,12], and fault detection [13]. The provided information can help APU maintenance in implementing repair and maintenance in a reasonable manner to save cost and enhance reliability [14]. 

Relative works in the PHM domain are mainly divided into the model-based method and the data-driven method [15,16]. The model-based method uses the laws of aerodynamics and thermodynamics of the APU to build a physical model, and this model can accurately describe its behavior. Combining this model with sensing data, its characteristics can be identified and PHM can be conducted. Due to the complex structure and unclear degradation mechanism of the APU, it is difficult to accurately formulate its physical model for predicting its performance sensing data. Different from the model-based method, the data-driven method mainly utilizes statistical theory and machine learning to achieve assessment results using the condition monitoring data. For data-driven methods, there is no need to know much knowledge about the mechanism of the physical object. As long as enough rich data are obtained, data-driven methods can be utilized to implement PHM. Thus, the data-driven method has become the research hotspot. Some research works have been carried out to evaluate the health status of the APU, including the statistical learning model, the hybrid model, and the artificial intelligence model.

The statistical learning model is mainly based on statistical knowledge. By using the related stochastic equations and practical parameters, the quantitative relationship of the condition monitoring data can be formulated as the parametric model. Zhang et al. [17] proposed the improved Weibull-based generalized renewal process model to predict the aircraft APU failure rate. Wang et al. [18] proposed an anomaly detection method based on a relevance vector machine to detect the anomalous exhaust gas temperature (EGT) data of the APU. Dong et al. [19] utilized the proportional hazards model to predict the RUL of the APU. Yang et al. [20] implemented APU starter prognostics using a particle filter-based method that is used to update the models of the performance monitoring parameters.

The hybrid model mainly refers to the integration of two or more different methods. The combination of these models makes use of the advantage of each method to make up for their deficiencies. In [21], a data-driven method based on the combination of mutual information and Gaussian process regression (GPR) was proposed to predict the RUL of the APU. A combination of GPR and ensemble empirical mode decomposition was studied in [22], which obtained the prior precision of its performance. Shetty et al. [23] proposed a hybrid prognostics model formulation system identification method to estimate the health status of the APU. Pascoal et al. [24] estimated the excessive bleed, compressor efficiency, and turbine efficiency loss via linear regression and neural networks. In [25], the physics-based model and neural network model were fused to monitor the starter degradation of the APU.

The artificial intelligence model allows the simulation of human intelligence or natural phenomena in machines to obtain more effective and accurate results. Vieira et al. [26] utilized the support vector classifier to process the peak of the exhaust temperature during the APU starting period. Then, its health state was analyzed from the starting process to the steady state. In view of the performance sensing data prediction of a complex system, such as the APU, they have nonlinear characteristics. The neural network [27] has potential capacity to realize the prediction of APU degradation, which has a strong nonlinear characterization ability. To solve the problem of the slow training of neural networks, an extreme learning machine (ELM) was proposed in the domain of machine learning [28].

Since the ELM was proposed, it has been widely utilized in several fields. In fact, the ELM is one kind of single-hidden layer feed forward neural network [29]. Compared with the traditional back propagation neural network algorithm [30], the ELM has the advantages of a faster learning speed and better generalization performance. In addition, it does not need to be adjusted during the training process. Only the number of neurons in the hidden layer need to be set to obtain the unique optimal solution. Thus, the ELM attracts the attention of scholars and has been successfully applied in many fields. Chaturvedi et al. [31] utilized an ELM to perform subjectivity detection. Cao et al. [32] proposed an enhanced ensemble-based ELM and sparse representation classification algorithm to realize image classification, which incorporates multiple ensembles to enhance the reliability of the classifier. Zhang et al. [33] utilized an ELM to improve the performance of the probabilistic semantic model.

However, the original ELM often leads to unstable prediction results [34]. In this study, the restricted Boltzmann machine (RBM) is adopted to optimize the ELM [35,36]. Then, a structurally stable ELM can be obtained. To evaluate the proposed method, the real sensing data from China Southern Airlines Company Limited Shenyang Maintenance Base (SYMOB) are adopted to carry out the evaluation experiments. Comparison experiments on three groups of data show that the optimized ELM has a more stable and better prediction performance.

The rest of this article is organized as follows. Section 2 introduces the proposed method and related theories. Section 3 presents experimental results and detailed discussion. Section 4 draws the conclusion and the future works.

## 2. Performance Sensing Data Prediction Using the Optimized ELM via the RBM

In this section, the proposed method is first illustrated. Then, the related theories such as the RBM and the ELM are presented in detail.

### 2.1. The Proposed Method

**Figure 1:**
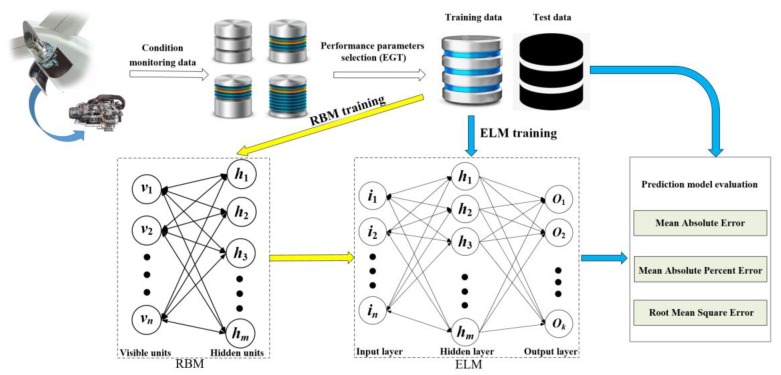
Diagram of the proposed method. EGT: exhaust gas temperature; RBM: restricted Boltzmann machine; ELM: extreme learning machine.

The ELM has the advantages of a faster learning speed and better generalization performance. To deal the problem of slow training of neural networks with the back propagation algorithm, an ELM was adopted to predict the performance sensing data of the APU in this study. However, the randomly generated weights and thresholds of the ELM often lead to unstable prediction results. To address this problem, an RBM was utilized to optimize the connection weights and the thresholds between the input layer and the hidden layer. In this way, a stable performance sensing data prediction model of the APU is expected to be achieved. The prediction results can also be obtained, and better prediction can be realized. The proposed method is illustrated in Figure 1.

To conduct the proposed method, the specific procedures are explained as follows.

Step 1. Choose the EGT data as the key parameter of the performance sensing data, which are from condition monitoring data of the APU. Preprocess the original data and divide the preprocessed data into training data and test data, respectively.

Step 2. Initiate the related parameters of the RBM and train the RBM with the available data.

Step 3. After the RBM is well trained, the weights and thresholds of the RBM are assigned to the ELM. 

Step 4. Utilize the training data to train the ELM. In this way, all parameters of the ELM can be determined.

Step 5. Predict the EGT of the next cycle based on the historical EGT. The predicted EGT data are used as part of the historical EGT. These combined data are utilized to predict the EGT of the next cycle. This process is repeated until the preset prediction steps are met.

Step 6. Evaluate the prediction results by appropriate metrics.

The error function in the ELM can be regarded as an energy function. In general, the optimal solution of network parameters can be obtained using the generalized inverse algorithm. However, if the weights and thresholds of the input layer and the hidden layer are generated randomly, the ELM network often falls into the local minimum point but fails to reach the global minimum point. The reason is that the error function or energy function of the network is a nonlinear space with multiple minimum points, and the random weight and threshold generated by the ELM will cause the network to fall into the local minimum value due to its randomness. Compared with the ELM, the main difference between a random network (e.g., RBM) and the ELM lies in the learning stage. Unlike other networks, a random network does not adjust weights based on a certain deterministic algorithm but modifies according to a certain probability distribution. In this way, the aforementioned defects can be effectively overcome. The net input of a neuron does not determine whether it is in a state of 1 or 0, but it does determine the probability that it is in a state of 1 or 0. This is the basic concept of a random neural network algorithm.

For the RBM network, with the evolution of the network state, the energy of the network always changes in the direction of decreasing in the sense of probability. This means that although the overall trend of the network energy is to evolve in the direction of a decrease, it cannot be excluded that some neuron states may have a value with a small probability. Thus, the network energy is increased temporarily. It is precise because of this possibility that the RBM network has the ability to jump out from the local minimum trough, which is the fundamental difference between the RBM and the ELM. This operation is called the search mechanism, which means that the network is in the process of running a continuous search for lower energy minima until the global minimum of the energy is achieved. 

### 2.2. Restricted Boltzmann Machine

**Figure 2:**
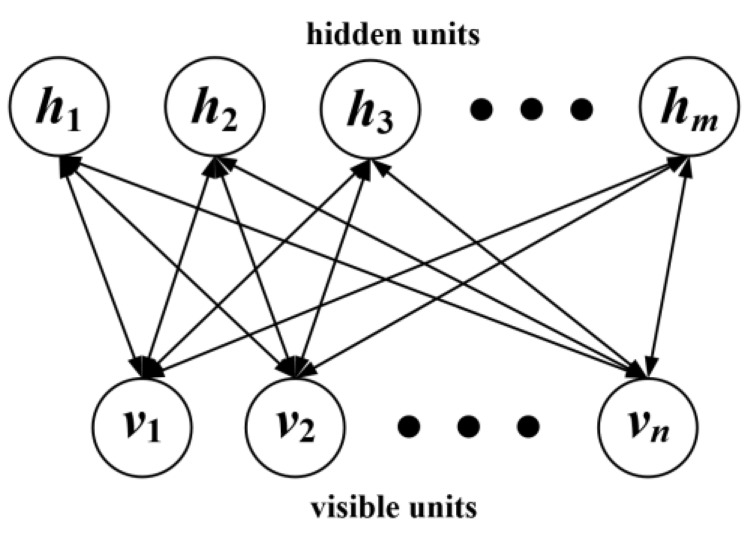
The structure of the RBM.

The RBM has only two layers of neurons, as shown in Figure 2. 

The first layer $v(v_{1},v_{2},\cdots ,v_{n})$ is named as the visible layer, which consists of visible units for training data input. The other layer $h(h_{1},h_{2},h_{3},\cdots ,h_{m})$ is named as the hidden layer, which consists of hidden units.

If the RBM includes *n* visible units and *m* hidden units, the vectors ***v*** and ***h*** can be used to represent the states of the visible and hidden units, respectively. $v_{i}$ denotes the state of the unit *i* in the visible layer and $h_{j}$ is the state of the unit *j* in the hidden layer. For the set (v,h), the energy of the RBM is defined by

(1) $$E(v,h|\theta )=-\sum_{i=1}^{n}a_{i}v_{i}-\sum_{j=1}^{m}b_{j}h_{j}-\sum_{i=1}^{n}\sum_{j=1}^{m}v_{i}W_{ij}h_{j},$$

where $\theta =\left\{W_{ij},a_{i},b_{j}\right\}$ is the parameter of the RBM, and $W_{ij}$ represents the connection weight between visible unit i and hidden unit j. $a_{i}$ is the bias of the visible unit i, and $b_{j}$ represents the bias of the hidden unit j. When the parameters are determined, the joint probability distribution of (v,h) can be obtained by

(2) $$P(v,h|\theta )=\frac{e^{-E(v,h|\theta )}}{Z(\theta )},$$

(3) $$Z(\theta )=\sum_{v,h}e^{-E(v,h|\theta )},$$

where Z(θ) is the normalized factor (also known as the partition function). The distribution P(v,θ) of observed data ***v*** defined by the RBM is the essential issue. To determine this distribution, the normalized factor Z(θ) needs to be calculated.

When the state of visible units in the RBM are given, the activation state of each hidden unit is conditionally independent. At this point, the activation probability of the unit *j* in the hidden layer is

(4) $$P(h_{j}=1|v,\theta )=\sigma \left(b_{j}+\sum_{i}v_{i}W_{ij}\right),$$

where σ is the sigmoid activation function. Since the structure of the RBM is symmetric, the activation state of each visible unit is also conditionally independent when the state of the hidden units is given. The activation probability of the unit *i* in the visible layer is

(5) $$P(v_{i}=1|h,\theta )=\sigma \left(a_{i}+\sum_{j}W_{ij}h_{j}\right).$$

It should be noted that there are no interconnections among the neurons in the visible and hidden layers. Only the inter-layer neurons have symmetrical lines and their relationship is independent, as given by

(6) $$P(h|v)=\prod_{j=1}^{N}P(h_{j}|v).$$

When the hidden layer is given, all explicit values are not related to each other, as illustrated by

(7) $$P(v|h)=\prod_{j=1}^{N}P(v_{j}|h).$$

With this property, it is not needed to calculate each neuron at every step. Instead, the neurons in the entire layer can be calculated by the parallel mode.

The training target of the RBM is to find the maximal probability distribution of hidden units with the training sample. Since the decisive factor lies in the weight *W*, the object of training the RBM is to determine the optimal weight.

The marginal distribution of joint probability distribution *P* is the likelihood function, as defined by

(8) $$\begin{array}{ll} P\left(\left.v\right|\theta \right) & =\sum_{h}p(v,h)=\frac{1}{Z(\theta )}\sum_{h}e^{-E(\left.v,h\right|\theta )}\\ \; & \;=\frac{1}{Z(\theta )}\sum_{h_{1}}\sum_{h_{2}}\cdots \sum_{h_{m}}e^{\sum_{i=1}^{n}a_{i}v_{i}}\prod_{j=1}^{m}e^{b_{j}h_{j}+\sum_{i=1}^{n}v_{i}W_{ij}h_{j}}\\ \; & \;=\frac{1}{Z(\theta )}e^{\sum_{i=1}^{n}a_{i}v_{i}}\sum_{h_{1}}e^{b_{1}h_{1}+\sum_{i=1}^{n}v_{i}W_{ij}h_{j}}\sum_{h_{2}}e^{b_{2}h_{2}+\sum_{i=1}^{n}v_{i}W_{ij}h_{j}}\cdots \sum_{h_{m}}e^{b_{m}h_{m}+\sum_{i=1}^{n}v_{i}W_{ij}h_{j}}\\ \; & \;=\frac{1}{Z(\theta )}\prod_{i=1}^{m}e^{a_{i}v_{i}}\prod_{j=1}^{m}(1+e^{b_{j}+\sum_{i=1}^{n}v_{i}W_{ij}}) \end{array}$$

When the training data *D* are given, the goal of training the RBM is to maximize the following likelihood:

(9) $$L_{\theta ,D}=\prod_{t=1}^{n}P(v^{t}).$$

Equation (9) can be equivalent to

(10) $$\ln L_{\theta ,D}=\ln\prod_{t=1}^{n}P(v^{t})=\sum_{t=1}^{n}\ln P(v^{t}).$$

A random gradient descent can be applied to solve the former problem. The derivative of lnP(v) with respect to θ needs to obtained, as illustrated by

(11) $$\begin{array}{ll} \frac{\partial \ln P(v)}{\partial \theta } & =\frac{\partial }{\partial \theta }\left(\ln\sum_{h}e^{-E(v,h)}\right)\;-\frac{\partial }{\partial \theta }\left(\ln\sum_{v,h}e^{-E(v,h)}\right)\\ & \;=-\frac{1}{\sum_{h}e^{-E(v,h)}}\sum_{h}e^{-E(v,h)}\frac{\partial E(v,h)}{\partial \theta }\;+\frac{1}{\sum_{v,h}e^{-E(v,h)}}\sum_{h}e^{-E(v,h)}\frac{\partial E(v,h)}{\partial \theta }\\ & \;=-\sum_{h}P(h|v)\frac{\partial E(v,h)}{\partial \theta }+\sum_{v,h}P(v,h)\frac{\partial E(v,h)}{\partial \theta }. \end{array}$$

The first term $\sum_{h}P(h|v)\frac{\partial E(v,h)}{\partial \theta }$ corresponds to the expectation of the energy gradient function $\frac{\partial E(v,h)}{\partial \theta }$ under the conditional distribution P(h|v). The second term $\sum_{v,h}P(v,h)\frac{\partial E(v,h)}{\partial \theta }$ corresponds to the expectation of the energy gradient function $\frac{\partial E(v,h)}{\partial \theta }$ under the joint distribution P(v,h).

The first term $\sum_{h}P(h|v)\frac{\partial E(v,h)}{\partial \theta }$ is easily calculated. However, P(v,h) represents the joint distribution of visible layer units and hidden layer units, which involves the normalized factor Z. This distribution is difficult to obtain. Therefore, we cannot calculate the second term, only its approximation can be achieved through some sampling methods.

Then, Equation (12) can be obtained, as given by

(12) $$\begin{array}{ll} \sum_{v,h}P(v,h)\frac{\partial E(v,h)}{\partial \theta } & =\sum_{v}\sum_{h}P(h|v)\frac{\partial E(v,h)}{\partial \theta }\\ & =\sum_{v}P(v)\sum_{h}P(h|v)\frac{\partial E(v,h)}{\partial \theta } \end{array}$$

When θ equals $W_{ij}$, we can get

(13) $$\begin{array}{ll} \sum_{h}P\left(\left.h\right|v\right)\frac{\partial E(v,h)}{\partial W_{ij}} & =-\sum_{h}P(\left.h\right|v)h_{i}v_{j}\\ \; & \;=-\sum_{h}\prod_{k=1}^{nh}P(\left.h_{k}\right|v)h_{i}v_{j}\\ \; & \;=-\sum_{h}P\left(\left.h_{i}\right|v\right)P\left(\left.h_{\_i}\right|v\right)h_{i}v_{j}\\ \; & \;=-\sum_{h_{i}}\sum_{h_{\_i}}P\left(\left.h_{i}\right|v\right)P\left(\left.h_{\_i}\right|v\right)h_{i}v_{j}\\ \; & \;=-\sum_{h_{i}}P(\left.h_{i}\right|v)h_{i}v_{j}\sum_{h_{\_i}}P(\left.h_{\_i}\right|v)\\ \; & \;=-\sum_{h_{i}}P(\left.h_{i}\right|v)h_{i}v_{j}=-P\left(\left.h_{i}=1\right|v\right)v_{j} \end{array}$$

When θ equals $a_{i}$, the following equation can be reached:

(14) $$\begin{array}{ll} \sum_{h}P(h|v)\frac{\partial E(v,h)}{\partial a_{i}} & =-\sum_{h}P(h|v)v_{i}\;=-v_{i}\sum_{h}P(h|v)\\ & =-v_{i} \end{array}$$

When θ equals $b_{i}$, Equation (15) can be achieved.

(15) $$\begin{array}{ll} \sum_{h}P\left(\left.h\right|v\right)\frac{\partial E(v,h)}{\partial b_{i}} & =-\sum_{h}P(\left.h\right|v){h_{i}}_{j}\\ \; & \;=-\sum_{h}\prod_{k=1}^{nh}P(\left.h_{k}\right|v)h_{i}\\ \; & \;=-\sum_{h}P\left(\left.h_{i}\right|v\right)P\left(\left.h_{\_i}\right|v\right)h_{i}\\ \; & \;=-\sum_{h_{i}}\sum_{h_{\_i}}P\left(\left.h_{i}\right|v\right)P\left(\left.h_{\_i}\right|v\right)h_{i}\\ \; & \;=-\sum_{h_{i}}P(\left.h_{i}\right|v)h_{i}\sum_{h_{\_i}}P(\left.h_{\_i}\right|v)\\ \; & \;=-\sum_{h_{i}}P(\left.h_{i}\right|v)h_{i}\\ \; & \;=-P(\left.h_{i}=1\right|v) \end{array}$$

Finally, the following three equations (Equations (16)–(18)) can be derived.

(16) $$\begin{array}{ll} \frac{\partial \ln P(v)}{\partial W_{ij}} & =-\sum_{h}P(h|v)\frac{\partial E(v,h)}{\partial W_{ij}}\;+\sum_{v,h}P(v,h)\frac{\partial E(v,h)}{\partial W_{ij}}\\ & \;=P(h_{i}=1|v)v_{j}-\sum_{v}P(v)P(h_{i}=1|v)v_{j} \end{array}$$

(17) $$\begin{array}{ll} \frac{\partial \ln P(v)}{\partial a_{i}} & =-\sum_{h}P(h|v)\frac{\partial E(v,h)}{\partial a_{i}}+\sum_{v,h}P(v,h)\frac{\partial E(v,h)}{\partial a_{i}}\\ & \;=v_{i}-\sum_{v}P(v)v_{i} \end{array}$$

(18) $$\begin{array}{ll} \frac{\partial \ln P(v)}{\partial b_{i}} & =-\sum_{h}P(h|v)\frac{\partial E(v,h)}{\partial b_{i}}+\sum_{v,h}P(v,h)\frac{\partial E(v,h)}{\partial b_{i}}\\ & \;=P(h_{i}=1|v)-\sum_{v}P(v)P(h_{i}=1|v) \end{array}$$

The computational complexity of $\sum_{h}$ in the above three equations is $2^{n+m}$. Therefore, the Markov chain Monte Carlo (MCMC) method, such as the Gibbs sampling method, is usually adopted for sampling, and uses samples to estimate $\sum_{h}$. However, each time MCMC sampling is performed, sufficient state transitions are required to ensure that the collected samples conform to the target distribution. It needs to collect a large number of samples accurately enough. These requirements greatly increase the complexity of RBM training. In this study, the contrastive divergence (CD) algorithm is adopted to obtain the parameters of the RBM. The *K* step CD algorithm is described as follows.

(19) $$\frac{\partial \ln P(v)}{\partial W_{ij}}\approx P(h_{i}=1|v^{\left(0\right)})v_{{}_{j}}^{\left(0\right)}-P(h_{i}=1|v^{\left(k\right)})v_{{}_{j}}^{\left(k\right)},$$

(20) $$\frac{\partial \ln P(v)}{\partial a_{i}}\approx v_{{}_{i}}^{\left(0\right)}-v_{{}_{i}}^{\left(k\right)},$$

(21) $$\frac{\partial \ln P(v)}{\partial b_{i}}\approx P(h_{i}=1|v^{\left(0\right)})-P(h_{i}=1|v^{\left(k\right)}).$$

**Figure 3:**
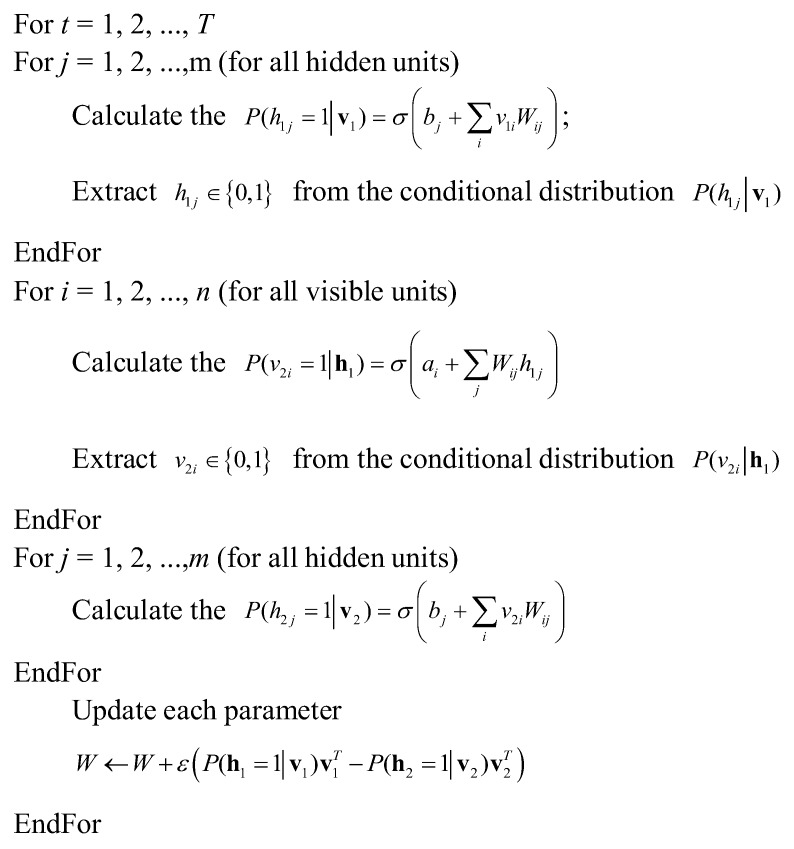
The training process of contrastive divergence (CD).

Let the connection weight matrix be *W*. The bias vector of the visible layer and the bias vector of the hidden layer are represented by *a* and *b*, respectively. The CD algorithm is shown in Figure 3.

After training, the RBM can accurately extract the features of the surface layer. Based on these features, the hidden layer can help in reconstructing the surface layer. As aforementioned, the original ELM performance is easily affected by the initialization of weights and thresholds. In this study, to solve this problem, the RBM was trained first. Then, the weights and thresholds of the trained RBM were transmitted to the ELM. In this way, an ELM with better performance can be obtained.

### 2.3. Extreme Learning Machine

**Figure 4:**
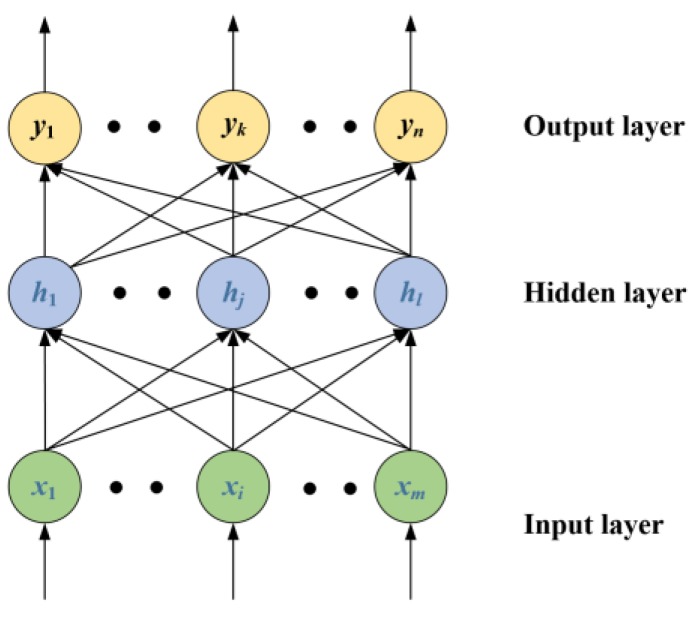
Single hidden layer neural network.

To solve the problem of the slow learning speed of traditional feed forward neural networks, Huang et al. [28] proposed a new learning algorithm, which is called the ELM. The structure of the ELM is the same as the traditional single hidden layer neural network, as shown in Figure 4.

In Figure 4, the input layer of the ELM contains *m* neurons, the hidden layer has *l* neurons, and the output layer contains *n* neurons.

Let $W_{i}$ and $b_{i}$ denote the connection weight and bias of the input layer and hidden layer. The output $y_{j}$ of the neural network is given by

(22) $$\sum_{j=1}^{l}\beta_{j}g(W_{j}X_{q}+b_{j})=y_{q},q=1,2,\ldots ,Q,$$

where *g*(*x*) is the activation function, $\beta_{j}$ is the output weight.

If there are *Q* arbitrary samples, the input matrix *X* and output matrix *Y* are expressed by

(23) $$X={\left[\begin{array}{cccc} x_{11} & x_{12} & \cdots & x_{mN}\\ x_{21} & x_{22} & \cdots & x_{mN}\\ \vdots & \vdots & \cdots & \vdots \\ x_{m1} & x_{m2} & \cdots & x_{mN} \end{array}\right]}_{m\times N},Y={\left[\begin{array}{cccc} y_{11} & y_{12} & \cdots & y_{1n}\\ y_{21} & y_{22} & \cdots & y_{2n}\\ \vdots & \vdots & \cdots & \vdots \\ y_{Q1} & y_{Q2} & \cdots & y_{Qn} \end{array}\right]}_{Q\times n}.$$

If the output matrix of the hidden layer is defined as *H*, then *H* can be given by

(24) $$H(W_{1},\ldots ,W_{l},b_{1},\ldots ,b_{l},X_{1},\ldots ,X_{Q})={\left[\begin{array}{ccc} g(W_{1}\cdot X_{1}+b_{1}) & \ldots & g(W_{l}\cdot X_{1}+b_{l})\\ \vdots & \ldots & \vdots \\ g(W_{1}\cdot X_{Q}+b_{1}) & \ldots & g(W_{l}\cdot X_{Q}+b_{l}) \end{array}\right]}_{Q\times l}.$$

Similarly, β can be expressed by

(25) $$\beta ={\left[\begin{array}{c} \beta_{1}^{T}\\ \vdots \\ \beta_{l}^{T} \end{array}\right]}_{l\times n}.$$

From Equation (22) to Equation (25), Equation (26) can be derived:

(26) Hβ=Y.

Let $\left(X_{q},t_{q}\right)$ denote an arbitrary sample, where the goal of the ELM is to minimize the output error, which is given by

(27) $$\min\sum_{q=1}^{Q}\left\|y_{q}-t_{q}\right\|.$$

Equation (27) can be transformed into

(28) $$\underset{\beta }{\min}\left\|H\beta =T\right\|.$$

By solving the least squares solution of Equation (28), the output weight β can be determined by

(29) $$\hat{\beta }=H^{†}T,$$

where $H^{†}$ is the Moore–Penrose generalized inverse of the matrix *H*.

## 3. Experimental Results and Discussion

To verify the effectiveness of the proposed method, the real APU sensing data from China Southern Airlines Company Limited Shenyang Maintenance Base were utilized. Three evaluation metrics were utilized to evaluate the performance sensing data prediction.

### 3.1. Data Description 

The condition monitoring data of the APU were from the aircraft communications addressing and reporting system, which mainly consist of four segments. They are the header, resume information, the operating parameters of the aircraft main engine, and the starting parameters of the APU. The header contains aircraft flight information, message generation, bleed valve status, opening angle, and total temperature. Resume information includes the APU serial number, operation hours, and number of cycles. The operating parameters are comprised of control command, exhaust gas temperature, guide vane opening angle, compressor inlet pressure, load compressor inlet port temperature, bleed air flow, bleed air pressure, oil temperature, and generator load. The starting parameters are made up of start-up interval, exhaust gas temperature peak, peak speed, oil temperature, and inlet temperature. Among the aforementioned parameters, EGT is the key performance parameter that can be utilized to predict APU degradation.

### 3.2. Evaluation Metrics

Let y(i) (i=1,2,…,N)  denote the real measured data and p(i) (i=1,2,…,N) refers to the predicted data. *N* indicates the number of predicted steps. The utilized metrics are given as follows.

(1) Mean absolute error (MAE)

(30) $$\mathrm{MAE}=\frac{1}{N}\sum_{i=1}^{N}\left|y(i)-p(i)\right|\;\;$$

In statistics, MAE is the quantity that can be used to measure how close the prediction data and the actual data are. A smaller value of MAE indicates a better accuracy of the prediction model.

(2) Mean absolute percent error (MAPE)

(31) $$\mathrm{MAPE}=\frac{1}{N}\sum_{i=1}^{N}\frac{\left|y(i)-p(i)\right|\;}{\left|y(i)\right|}\;$$

MAPE is adopted to measure the intuitive interpretation in the prospect of relative error.

(3) Root mean square error (RMSE)

(32) $$\mathrm{RMSE}=\sqrt{\sum_{i=1}^{n}{\left(y(i)-p(i)\right)}^{2}/N}$$

RMSE represents the expected data of the squared error. A smaller RMSE value denotes better stability of the prediction model.

### 3.3. Experimental Results

In this section, the comparison experiments between the original ELM and the proposed method are carried out. To fully measure the effectiveness of the proposed method, three groups of experiments with different training data were conducted.

The number of training data points varies from 240 to 320, while the number of test data points varies from 21 to 41 for prediction accuracy assessment. The details of these three datasets are shown in Table 1.

#### 3.3.1. Experiments Implemented with the ELM

**Figure 5:**
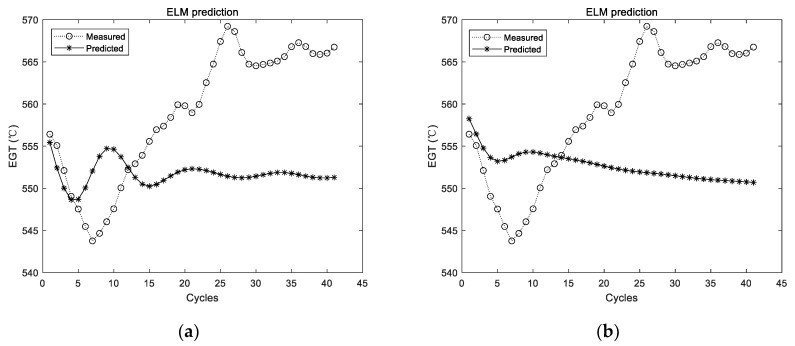
Experimental results of ELM prediction using the first group of data. (**a**) The first experimental results; (**b**) the second experimental results.

The utilized ELM in those experiments had a hidden layer, which consisted of 20 ELM neurons. The ELM was firstly trained with the training data. Then, the trained ELM was adopted for prediction. The prediction results of the first group of data are shown in Figure 5.

**Figure 6:**
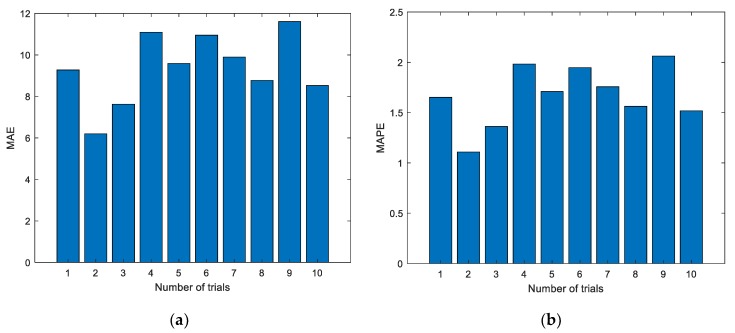

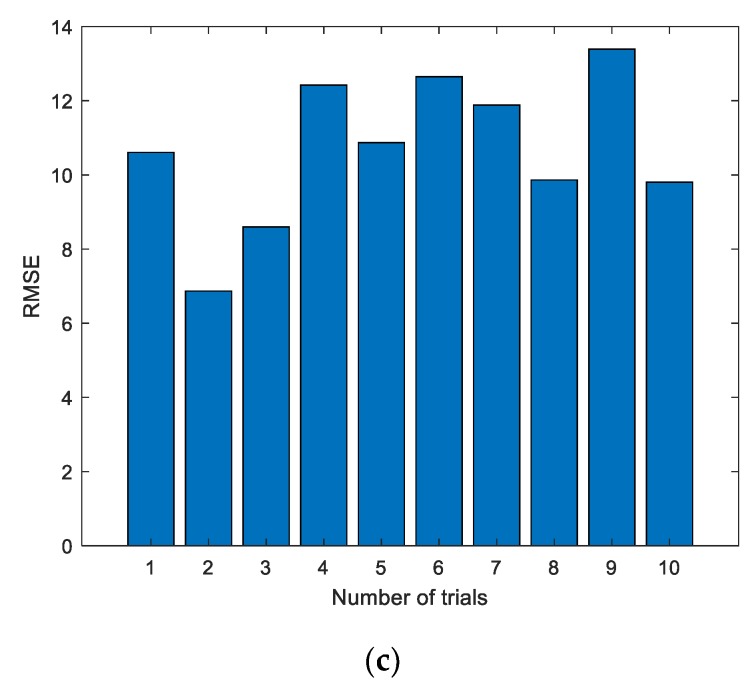
Evaluation metrics of ELM prediction using the first group of data. (**a**) Mean absolute error (MAE); (**b**) mean absolute percent error (MAPE); (**c**) root mean square error (RMSE).

Figure 5 shows the experimental results with the data of the first group. The experiment was conducted for a total of 10 times, and the results of the first two experiments are shown in Figure 5; the dotted line represents the actual measured EGT, and the starred line represents the predicted EGT. It can be intuitively seen that the prediction results of the ELM are unstable. In addition, the prediction deviation is relatively larger when the number of prediction steps increases. The detailed evaluation metrics of ELM prediction utilizing the first group of data are illustrated in Figure 6.

Figure 6 shows the evaluation metrics of the experiments. As shown in the charts, each experiment produces a unique prediction result. To further explore the prediction performance of the ELM, experiments were conducted 10, 20, 50, 100, and 200 times, respectively. The details of the experimental results are listed in Table 2.

**Figure 7:**
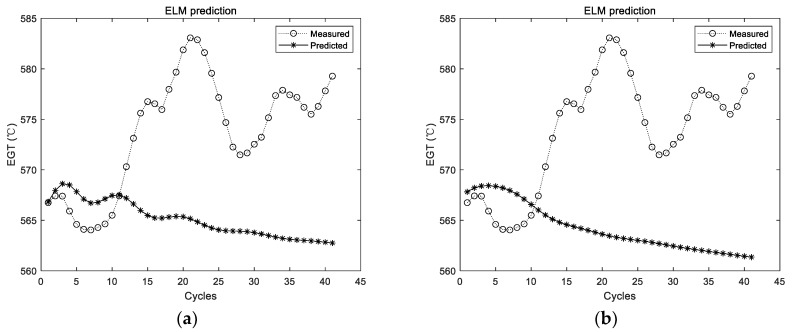
Experimental results of ELM prediction using the second group of data. (**a**) The first experimental results; (**b**) the second experimental results.

**Figure 8:**
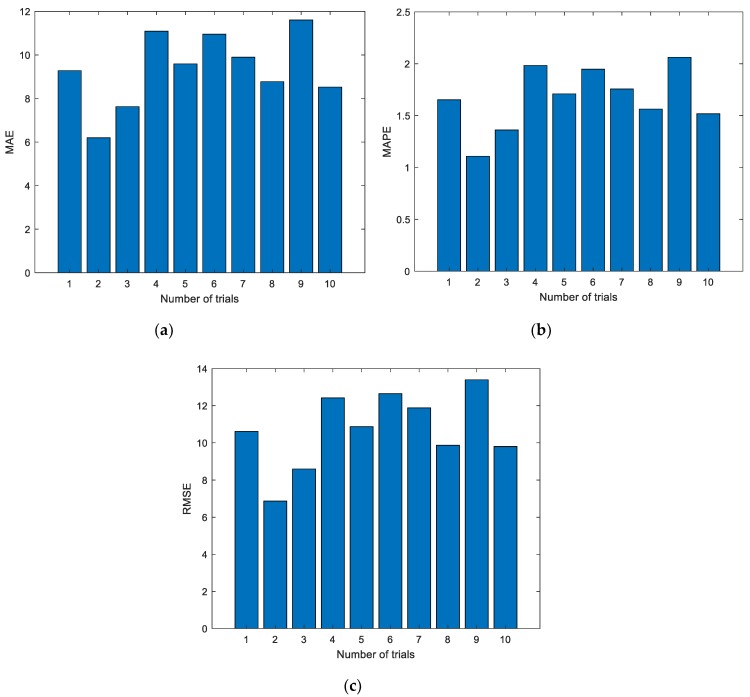
Evaluation metrics of ELM prediction using the second group of data. (**a**) MAE; (**b**) MAPE; (**c**) RMSE.

Experimental results with the data of the second group are shown in Figure 7 and Figure 8. The experimental setting conditions are the same as the previous experiments.

**Figure 9:**
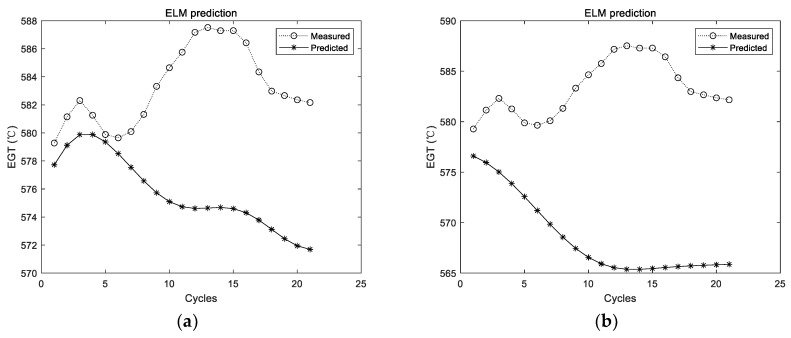
Experimental results of ELM prediction using the third group of data. (**a**) The first experimental results; (**b**) the second experimental results.

**Figure 10:**
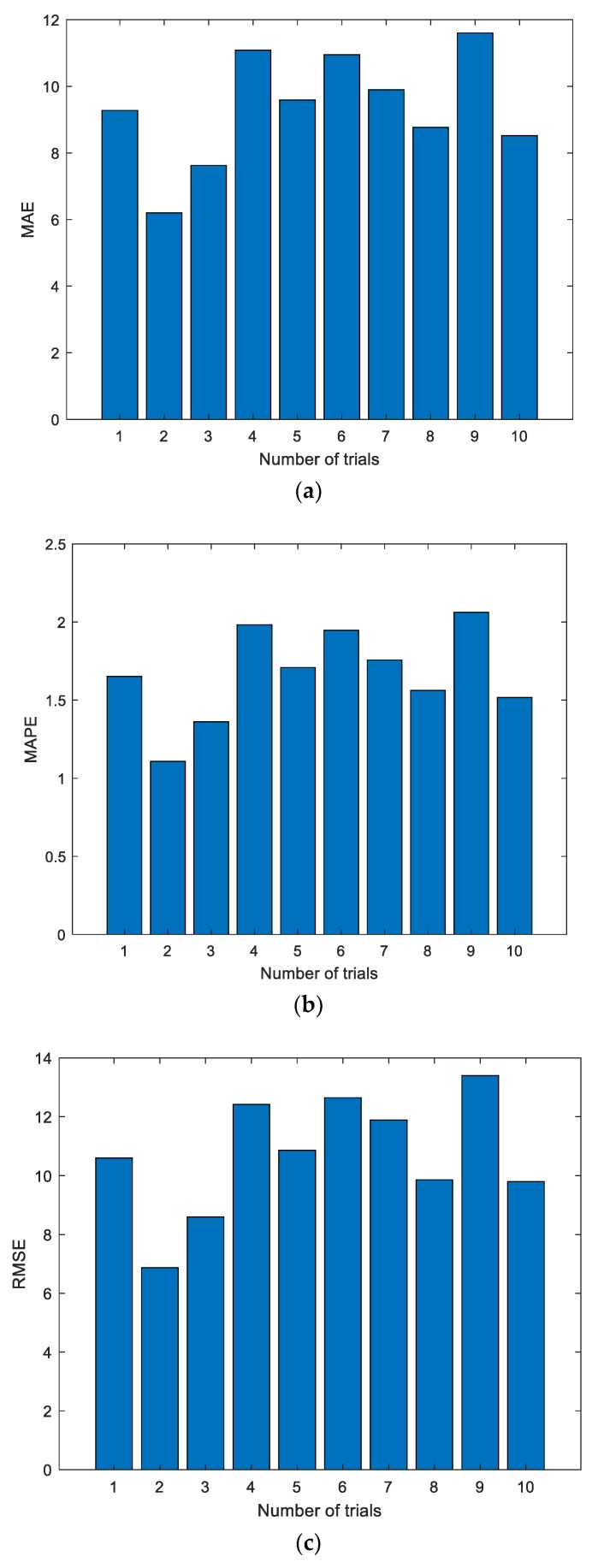
Evaluation metrics of ELM prediction using the third group of data. (**a**) MAE; (**b**) MAPE; (**c**) RMSE.

Experimental results with the data of the third group are shown in Figure 9 and Figure 10. The experimental setting conditions are the same as the previous experiments.

In the figures and tables above, Figure 5, Figure 7 and Figure 9 show the experimental results with the data of Group 1, Group 2, and Group 3, respectively. Similarly, Figure 6, Figure 8 and Figure 10 show the evaluation metrics. Metrics of comparison experiments are summarized in Table 2, Table 3 and Table 4.

#### 3.3.2. Experiments Implemented with the Proposed Method

**Figure 11:**
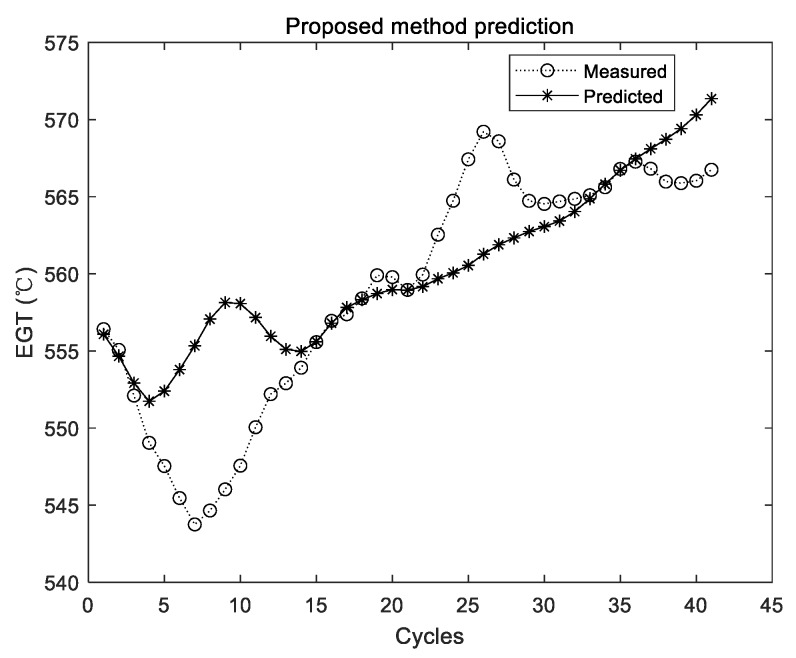
Experimental results of the proposed method using the first group of data.

**Figure 12:**
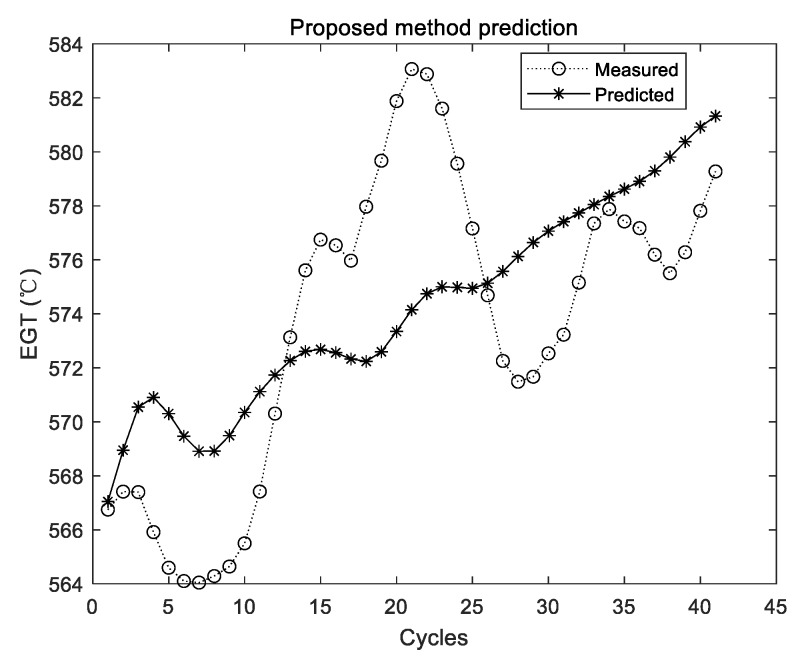
Experimental results of the proposed method using the second group of data.

**Figure 13:**
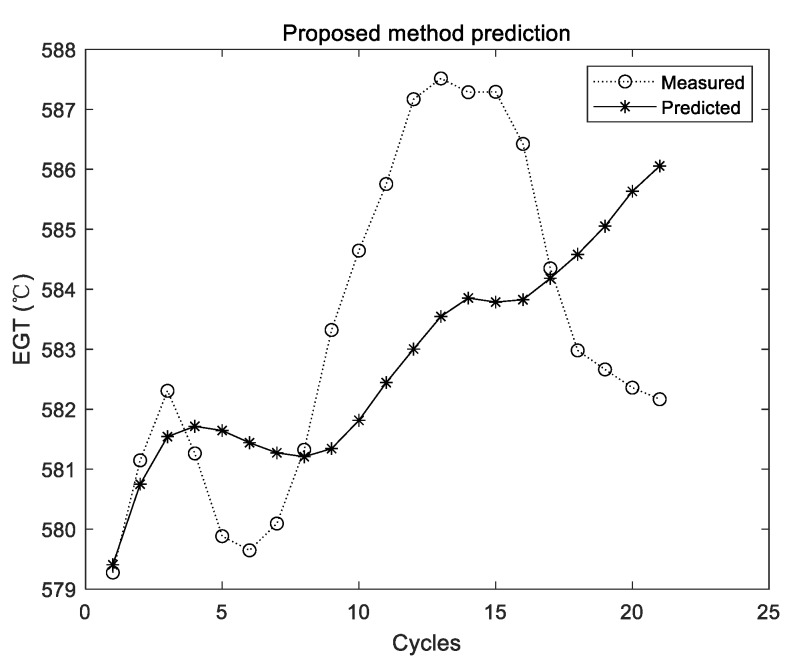
Experimental results of the proposed method using the third group of data.

The prediction results of the proposed method are shown in Figure 11, Figure 12 and Figure 13.

The two curves in Figure 11, Figure 12 and Figure 13 have the same meaning as those in Figure 5, Figure 7 and Figure 9. It can be seen that the two curves are close to each other, which denotes that the predicted EGT data are more precise. As shown in Section 3.3.1, there is no evidence shown that more experiments make better predictions on average. Thus, in the comparison experiments, the average results of 10 times are adopted. Results of comparison experiments are given in Table 5, Table 6 and Table 7.

### 3.4. Discussion

Since the prediction results of the ELM were not stable, the ELM was conducted for 10, 20, 50, 100, and 200 times, respectively, in each group of experiments. The average results of 10 times were considered as the comparison experiment. ELM prediction results for each group of experiments are randomly listed in Figure 5, Figure 7 and Figure 9, respectively. It can be seen intuitively from the figures that the randomly initialized ELM prediction results are not stable, and also fail to predict the degradation trend of the APU. 

As can be seen from Figure 6, Figure 8 and Figure 10, the evaluation indexes of each experiment are different. The experiments also confirm that the random initialization of weights and thresholds will lead to unstable ELM prediction results.

From Table 2 to Table 4, the evaluation metrics of each group under different experiment times are given. In fact, this method belongs to ensemble learning. By using the ensemble learning approach, more stable prediction results can be obtained. However, in Table 2, when the number of experiments is 50, the prediction results are the worst, in which MAE, MAPE, and RMSE are 10.2111, 1.8165, and 11.8673, respectively. When the experiment number is set as 50, the best prediction results can be obtained, in which the MAE, MAPE, and RMSE are 9.2661, 1.6488, and 10.7172, respectively. While in Table 3, when the number of experiments is 10, the prediction results are the worst, in which MAE, MAPE, and RMSE are 14.8342, 2.5747, and 16.4750, respectively. When the experiment number is set as 20, the best prediction results can be obtained, in which the smallest MAE, MAPE, and RMSE are 14.0302, 2.4360, and 15.6901, respectively. As shown in Table 4, when the number of experiments is 100, the prediction results are the worst, in which MAE, MAPE, and RMSE are 11.4609, 1.9621, and 13.1249, respectively. When the experiment number is set as 20, the best prediction results can be obtained, in which the smallest MAE, MAPE, and RMSE are 8.9778, 1.5366, and 10.2694, respectively. Experimental results show that the prediction results will not necessarily be improved as the number of experiments increases. The reason is that the prediction results of the ELM are not stable.

As shown from Table 5 to Table 7, although experimental results of the original ELM have been integrated and the averaged value is adopted, the values of the three metrics are all larger than those of the proposed method. By using the proposed method, MAE, MAPE, and RMSE have declined to 35.7%, 36.1%, and 45.7% of their initial value in Group 1, respectively. In the second group, MAE, MAPE, and RMSE have declined to 27.5%, 27.6%, and 28.1% of their initial value, respectively. In the third group, MAE, MAPE, and RMSE have declined to 21.9%, 21.9%, and 23.8% of their initial value, respectively. Therefore, compared with the original ELM, the proposed method can obtain better results of performance sensing data prediction. To be specific, the accuracy, relative error, and stability of the proposed method are all relatively better.

## 4. Conclusions

In this article, we studied the performance sensing data prediction of an aircraft APU. To utilize the nonlinear features contained in these data, the optimized ELM using an RBM was proposed. In this way, the relatively appropriate weights and thresholds of the ELM for implementing prediction can be determined. Three kinds of evaluation experiments were implemented in different lengths of training data and test data. Compared with the original ELM, the proposed method can achieve better accuracy and stable prediction results. Therefore, the proposed method not only provides one feasible way for predicting the performance sensing data that can be regarded as an indicator of the health condition of the APU, but also offers the idea for improving the ELM. However, the influence of environmental factors (e.g., ambient temperature and atmospheric pressure) on EGT sensing data was not considered. We will take the physical model of the bleed air performance to modify EGT data. It is expected to enhance the prediction results even further.

## Figures and Tables

**Table 1:** Dataset details of comparison experiments.

Group Number	Group 1	Group 2	Group 3
Number of training data points	240	280	320
Number of test data points	41	41	21

**Table 2:** Metrics of comparison experiments (Group 1).

Experimental Number	MAE	MAPE	RMSE
10	9.3887	1.6708	10.7473
20	9.2661	1.6488	10.7172
50	10.2111	1.8165	11.8673
100	9.6952	1.7246	11.2039
200	9.6640	1.7194	11.3328

**Table 3:** Metrics of comparison experiments (Group 2).

Experimental Number	MAE	MAPE	RMSE
10	14.8342	2.5747	16.4750
20	14.0302	2.4360	15.6901
50	14.7773	2.5652	16.5192
100	14.2241	2.4687	16.0747
200	11.3842	2.0238	14.1186

**Table 4:** Metrics of comparison experiments (Group 3).

Experimental Number	MAE	MAPE	RMSE
10	9.4750	1.6215	10.4293
20	8.9778	1.5366	10.2694
50	9.5006	1.6263	10.5143
100	11.4609	1.9621	13.1249
200	9.9503	1.7033	10.9661

**Table 5:** Metrics of comparison experiments (Group 1).

Method	MAE	MAPE	RMSE
The original ELM	9.3887	1.6708	10.7473
The proposed method	3.3471	0.6030	4.9124

**Table 6:** Metrics of comparison experiments (Group 2).

Method	MAE	MAPE	RMSE
The original ELM	14.0302	2.4360	15.6901
The proposed method	3.8569	0.6718	4.4142

**Table 7:** Metrics of comparison experiments (Group 3).

Method	MAE	MAPE	RMSE
The original ELM	9.4750	1.6215	10.4293
The proposed method	2.0815	0.3561	2.4851

## References

[1] McCown P.M., Conway T.J., Conway C.V. Auxiliary power unit maintenance aid-flight line engine diagnostics. Proceedings of the IEEE Automatic Testing Conference. The Systems Readiness Technology Conference. Automatic Testing in the Next Decade and the 21st Century. Conference Record.

[2] Chen X., Lyu Z., Ren H., Wang H., Li L., Huang J., Chen Y. APU feature integration based on multi-variant flight data analysis. Proceedings of the 2016 IEEE International Conference on Prognostics and Health Management (ICPHM).

[3] Yang C., Létourneau S., Yang Y., Liu J. Data mining based fault isolation with FMEA rank: A case study of APU fault identification. Proceedings of the 2013 IEEE Conference on Prognostics and Health Management (PHM).

[4] Letourneau S., Yang C., Liu Z. Improving preciseness of time to failure predictions: Application to APU starter. Proceedings of the 2008 International Conference on Prognostics and Health Management.

[5] Yabsley A., Ibrahim Y. Study on maintenance contribution to life cycle costs: Aircraft auxiliary power unit example. Proceedings of the 2008 IEEE International Conference on Industrial Technology.

[6] Liu L., Liu D., Zhang Y., Peng Y. (2016). Effective sensor selection and data anomaly detection for condition monitoring of aircraft engines. Sensors.

[7] Liu L., Pan D., Liu D., Zhang Y., Peng Y. (2016). DRES: Data recovery for condition monitoring to enhance system reliability. Microelectron. Reliab..

[8] Kushan M.C., Peng Z.X., Peng S.Z. (2012). A New System of Failure Warning and Monitoring Control System for Gas Turbine Compressor (GTC)/Auxiliary Power Unit (APU) of Aircrafts and a Sample Study. Adv. Mater. Res..

[9] Liu L., Liu M., Guo Q., Liu D., Peng Y. MEMS Sensor data anomaly detection for the UAV flight control subsystem. Proceedings of the 2018 IEEE SENSORS.

[10] Duan Y., Zhao Y., Pang J., Liu L., Liu D. Unmanned Aerial Vehicle Sensing Data Anomaly Detection by Relevance Vector Machine. Proceedings of the 2017 International Conference on Sensing, Diagnostics, Prognostics, and Control (SDPC).

[11] Liu L., Guo Q., Liu D., Peng Y. (2019). Data-Driven Remaining Useful Life Prediction Considering Sensor Anomaly Detection and Data Recovery. IEEE Access.

[12] Liu L., Wang S., Liu D., Peng Y. (2017). Quantitative selection of sensor data based on improved permutation entropy for system remaining useful life prediction. Microelectron. Reliab..

[13] Lou Q. (2013). Aircraft APU Starter Health Monitoring and Failure Prognostics. Ph.D. Thesis.

[14] Yang C., Letourneau S., Liu J., Cheng Q., Yang Y. (2017). Machine learning-based methods for TTF estimation with application to APU prognostics. Appl. Intell..

[15] Gorinevsky D., Dittmar K., Mylaraswamy D., Nwadiogbu E. Model-based diagnostics for an aircraft auxiliary power unit. Proceedings of the International Conference on Control Applications.

[16] Liu L., Peng Y., Liu D. (2016). FESeR: A data-driven framework to enhance sensor reliability for the system condition monitoring. Microelectron. Reliab..

[17] Zhang Y., Peng Y., Wang P., Wang L., Wang S., Liao H. Aircraft APU failure rate prediction based on improved Weibull-based GRP. Proceedings of the 2017 Prognostics and System Health Management Conference (PHM-Harbin).

[18] Wang L., Li M., Liu L., Liu D. Exhaust gas temperature sensing data anomaly detection for aircraft auxiliary power unit condition monitoring. Proceedings of the 2018 International Conference on Sensing, Diagnostics, Prognostics, and Control (SDPC).

[19] Dong P., Cai J., Zhang L. Residual Life Prediction of Aviation APU Based on PH Model. Proceedings of the 2017 International Conference on Sensing, Diagnostics, Prognostics, and Control (SDPC).

[20] Yang C., Lou Q., Liu J., Yang Y., Bai Y. Particle filter-based method for prognostics with application to auxiliary power unit. Proceedings of the International Conference on Industrial, Engineering and Other Applications of Applied Intelligent Systems.

[21] Liu L., Wang L., Wang S., Liu D., Peng Y. Remaining Useful Life Prediction of Aircraft Auxiliary Power Unit with On-wing Sensing Data. Proceedings of the 2018 Prognostics and System Health Management Conference (PHM-Chongqing).

[22] Chen X., Wang H., Huang J., Ren H. APU degradation prediction based on EEMD and Gaussian process regression. Proceedings of the 2017 International Conference on Sensing, Diagnostics, Prognostics, and Control (SDPC).

[23] Shetty P., Mylaraswamy D., Ekambaram T. A hybrid prognostic model formulation system identification and health estimation of auxiliary power units. Proceedings of the 2006 IEEE Aerospace Conference.

[24] Pascoal R.M., Vianna W.O., Gomes J.P., Galvão R.K. Estimation of APU failure parameters employing linear regression and neural networks. Proceedings of the Annual Conference of the Prognostics and Health Management Society.

[25] Zhang Y., Liu J., Hanachi H., Yu X., Yang Y. Physics-based Model and Neural Network Model for Monitoring Starter Degradation of APU. Proceedings of the 2018 IEEE International Conference on Prognostics and Health Management (ICPHM).

[26] Vieira F.M., de Oliveira Bizarria C., Nascimento C.L., Fitzgibbon K.T. Health monitoring using support vector classification on an auxiliary power unit. Proceedings of the 2009 IEEE Aerospace Conference.

[27] Wang B., Wang Z., Liu L., Liu D., Peng X. Data-Driven Anomaly Detection for UAV Sensor Data Based on Deep Learning Prediction Model. Proceedings of the 2019 Prognostics and System Health Management Conference (PHM-Paris).

[28] Huang G.-B., Zhu Q.-Y., Siew C.-K. (2004). Extreme learning machine: A new learning scheme of feedforward neural networks. Neural Netw..

[29] Huang G.-B., Wang D.H., Lan Y. (2011). Extreme learning machines: A survey. Int. J. Mach. Learn. Cybern..

[30] Mizutani E., Dreyfus S.E., Nishio K. On derivation of MLP backpropagation from the Kelley-Bryson optimal-control gradient formula and its application. Proceedings of the IEEE-INNS-ENNS International Joint Conference on Neural Networks. IJCNN 2000. Neural Computing: New Challenges and Perspectives for the New Millennium.

[31] Chaturvedi I., Ragusa E., Gastaldo P., Zunino R., Cambria E. (2018). Bayesian network based extreme learning machine for subjectivity detection. J. Frankl. Inst..

[32] Cao J., Hao J., Lai X., Vong C.-M., Luo M. (2016). Ensemble extreme learning machine and sparse representation classification. J. Frankl. Inst..

[33] Zhang Y., Yuan Y., Guo F., Wang Y., Wang G. (2018). Improving the multimodal probabilistic semantic model by ELM classifiers. J. Frankl. Inst..

[34] Neumann K., Steil J.J. (2013). Optimizing extreme learning machines via ridge regression and batch intrinsic plasticity. Neurocomputing.

[35] Hinton G.E., Osindero S., Teh Y.-W. (2006). A fast learning algorithm for deep belief nets. Neural Comput..

[36] Mukherjee H., Obaidullah S.M., Santosh K., Phadikar S., Roy K. (2018). Line spectral frequency-based features and extreme learning machine for voice activity detection from audio signal. Int. J. Speech Technol..

